# Minimal within-host dengue models highlight the specific roles of the immune response in primary and secondary dengue infections

**DOI:** 10.1098/rsif.2014.0886

**Published:** 2015-02-06

**Authors:** Rotem Ben-Shachar, Katia Koelle

**Affiliations:** 1 Program in Computational Biology and Bioinformatics, Duke University, Durham, NC, USA; 2 Department of Biology, Duke University, Durham, NC, USA; 3 Fogarty International Center, National Institute of Health, Bethesda, MD, USA

**Keywords:** dengue, mathematical model, viral dynamics, immune response, cytokine storm, disease severity

## Abstract

In recent years, the within-host viral dynamics of dengue infections have been increasingly characterized, and the relationship between aspects of these dynamics and the manifestation of severe disease has been increasingly probed. Despite this progress, there are few mathematical models of within-host dengue dynamics, and the ones that exist focus primarily on the general role of immune cells in the clearance of infected cells, while neglecting other components of the immune response in limiting viraemia. Here, by considering a suite of mathematical within-host dengue models of increasing complexity, we aim to isolate the critical components of the innate and the adaptive immune response that suffice in the reproduction of several well-characterized features of primary and secondary dengue infections. By building up from a simple target cell limited model, we show that only the innate immune response is needed to recover the characteristic features of a primary symptomatic dengue infection, while a higher rate of viral infectivity (indicative of antibody-dependent enhancement) and infected cell clearance by T cells are further needed to recover the characteristic features of a secondary dengue infection. We show that these minimal models can reproduce the increased risk of disease associated with secondary heterologous infections that arises as a result of a cytokine storm, and, further, that they are consistent with virological indicators that predict the onset of severe disease, such as the magnitude of peak viraemia, time to peak viral load, and viral clearance rate. Finally, we show that the effectiveness of these virological indicators to predict the onset of severe disease depends on the contribution of T cells in fuelling the cytokine storm.

## 1. Introduction

Dengue is estimated to infect 390 million individuals annually [1], making it the most prevalent arthropod-borne viral disease in the world. The virus is classified into four related, but distinct, serotypes, DENV-1 through DENV-4, each of which is transmitted between humans primarily by the mosquito *Aedes aegypti*. While most cases are asymptomatic, disease symptoms, occurring in approximately one quarter of dengue infections [1], range in severity from ‘breakbone’ dengue fever (DF) to life-threatening dengue haemorrhagic fever (DHF), which is characterized by vascular plasma leakage and may lead to circulatory shock known as dengue shock syndrome (DSS) [2].

These more severe clinical manifestations of dengue have prompted numerous studies aimed at improving our understanding of dengue pathogenesis, and with it, the role that the immune response plays in controlling viral infection. Experimental studies in mice have shown that the innate immune response is important for clearing a primary dengue infection, whereas the adaptive immune response contributes to the development of severe disease [3,4]. Other studies have shown that human dengue antibodies can enhance viral growth *in vitro* and thereby increase the risk of developing severe disease in a secondary infection with a heterologous serotype [5,6]. Further studies have shown that memory T-cells established during a primary infection may act to increase the risk of developing severe disease in a heterologous secondary infection through increased pro-inflammatory cytokine production [7,8]. Complementing these experimental studies, epidemiological studies have successfully isolated host and viral risk factors associated with severe disease [9–12]. Taken together, these studies have indicated that excessive activation of the immune response during a dengue infection may lead to a cascade of cytokine production, known as a cytokine storm, that results in direct damage to vascular endothelial cells and increased capillary permeability [7,13,14]. This cytokine storm phenomenon is not unique to dengue, having also been used to describe pathologies resulting from other viral infections including influenza, cytomegalovirus and severe acute respiratory syndrome coronavirus [13].

Apart from experimental studies of viral pathogens, mathematical models describing infection dynamics within hosts have provided additional insights into viral kinetics and disease outcomes. These models have in large part focused on chronic infectious diseases, such as human immunodeficiency virus (HIV) [15,16] and hepatitis C virus [15,17]. For diseases causing acute infection, influenza has been the most extensively studied pathogen to date, probably due to the availability of human and non-human animal challenge study data. These influenza models have highlighted the importance of both the innate and the adaptive immune response in regulating viral dynamics [18–21], and particularly, the role of the innate immune response in contributing to disease symptoms [20,22].

For dengue, we are aware of four existing within-host models. Three of these models consider the dynamic interaction between free virus, uninfected target cells, infected target cells and immune cells [23–25], differing from one another only in the functional forms used to model viral infectivity, viral clearance and immune cell dynamics. In all three of these models, the immune cells play a protective role by clearing infected cells and are therefore likely to represent T cells. None of these models considers the known effects that T cells and more generally, the adaptive immune response may have in contributing to dengue disease. Of note, one of these models [25] was statistically fit to individual-level patient data, with findings indicating that differences in viral dynamics between primary and secondary infections can be recovered by a higher viral infectivity rate during secondary infections. This result is consistent with evidence for the enhancement of viral infectivity as a result of increased levels of non-neutralizing antibodies during a secondary infection relative to a primary infection. The fourth model considers the dynamic interaction between free virus, uninfected cells, infected cells, B cells and antibodies [26]. In this model, the effect of antibodies is either protective or enhancing, depending on the antigenic similarity between the virus of the primary infection and the virus of the secondary infection. However, this model does not provide an explicit mechanism by which disease arises. Instead, it assumes that disease severity is positively correlated with the level of antibodies in a heterologous secondary infection. None of these existing dengue models consider the role that the innate immune response plays in controlling the viral infection or how infection dynamics explicitly impact disease severity.

Here, we aim to develop minimal within-host mathematical models of dengue infection that are able to recover known features of primary and secondary dengue infections, and that can be used as a basis for understanding characterized patterns of disease severity. Our approach is thus to analyse models of increasing complexity, evaluating each model by the dynamic features it can yield. We begin with a simple target cell limited model, and in a step-wise fashion, consider the dynamical effects that the innate and the adaptive immune responses contribute to dengue infection dynamics. By first parametrizing the considered models for a primary symptomatic infection using a combination of literature estimates and clinical observations that serve as constraints on model parameters, we show that only the innate immune response is needed to recover the characteristic features of a primary infection. To arrive at a minimal model for a secondary dengue infection, we build up from the minimal primary infection model to recover the characteristic features of a secondary infection that differ from those of a primary infection. We find that higher viral infectivity rates along with infected cell clearance by T cells are necessary to reproduce these characteristic features of a secondary dengue infection, underscoring the contribution of the adaptive immune response in these infections. We further show that the minimal dengue models selected in our analysis yield viral dynamics consistent with recently published individual viral load data for dengue [25].

While it has been established that higher viral loads are associated with severe disease, there is discordance in the dengue literature on whether the cellular immune response is protective or facilitates the onset of severe disease [7,14,27,28]. With the risk of developing severe disease depending on circulating cytokine levels, we develop two disease severity parametrizations that differ in the contribution of T-cell-secreted cytokines to disease severity. To discriminate between these different formulations, we then examine the relationship between known virological indicators of disease severity that have been isolated in the clinical literature and the risk of developing severe disease.

The models we present here are the first, to the best of our knowledge, to quantitatively describe how the interaction of the immune system with dengue virus can lead to increased cytokine production and thereby an increased risk of manifesting severe disease. The analysis of the virological indicators of disease severity provides insight into how infected cells and T cells may contribute to disease severity. Furthermore, our analysis highlights how the effectiveness of virological indicators in predicting dengue disease severity critically depends on the contribution of T cells to disease severity.

## 2. Selection of minimal dengue models

### 2.1. Characteristic features of a primary dengue infection

Viral kinetic data from dengue patients are often absent prior to fever onset (e.g. [9–11,29]). A comprehensive study of cytokines from human infections are similarly missing [30,31], or are taken at few or even only a single time point post-infection [32,33]. Furthermore, there is considerable variation among studies in cytokine levels, potentially due to their relatively short half-lives [34]. Instead of evaluating different mathematical models directly by their fit to these incomplete (if at all available) data, we here first develop a minimal model for a primary dengue infection that is able to reproduce well-established characteristic features of the viral infection.

For a primary dengue infection resulting in DF, these features are (i) target cells (monocytes [14]) deplete to approximately 60% of their original level during dengue infection (from approx. 3.5 × 10^5^ cells ml^−1^ to 2 × 10^5^ cells ml^−1^) [12]; (ii) peak viraemia occurs approximately 9 days post viral inoculation based on estimates of the incubation period [35] and the timing of peak viraemia after the onset of symptoms [9]; (iii) maximum daily clearance rate is approximately 2.2 log_10_ copies per millilitre per day [9], and (iv) peak viraemia is approximately 9.7 log_10_ copies per millilitre. The latter two features are measured from DENV-1 patients [9], so may not perfectly reflect the characteristic features of dengue serotypes 2–4.

### 2.2. Target cell limited model

We begin by examining a target cell limited model, conceivably the simplest within-host model formulation. This model, previously used in a model of acute influenza infection [36], is given by
2.1
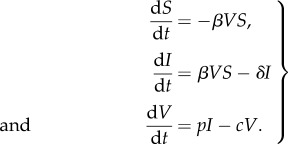

Susceptible cells (*S*) are depleted by becoming infected with free virus (*V*) at rate *βVS*. Free virus is produced by infected cells (*I*) at rate *pI* and cleared at rate *cV*. Infected cells die at rate *δI*. We can further reduce the system of equations to two dimensions by assuming, consistent with empirical findings [37], that the dynamics of the virus are fast relative to the dynamics of infected cells. With this quasi-steady-state assumption, equations (2.1) become
2.2
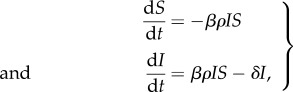

with *V*(*t*) = *ρI*(*t*) and *ρ* = *p*/*c*. Given that we expect a maximal 2.2 log_10_ copies per millilitre daily decline in viral clearance (the third characteristic feature listed above), we can calculate the minimum *δ* needed to achieve this viral decline by assuming that *S* ≈ 0 for a short time interval after the viral peak. This assumption was also used to approximate the viral decline rate after viral peak in [21]. Under this assumption, d*I*/d*t* = −*δI* and 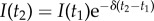, where *t*_2_ = *t*_1_ + 1. Using the approximation *V* = *ρI*, *V*(*t*_2_)/*V*(*t*_1_) = e^−^^*δ*^. To achieve a 2.2 log_10_ viral decline, the minimum *δ* needed is 5.1 per day.

To determine whether this estimate is biologically reasonable, we first note that the dengue virus life cycle results in exocytosis of mature infectious particles from the infected cell, and therefore does not result in bursting of infected cells [38]. Dengue virus has also been shown to be able to manipulate the antiviral response, resulting in delay or prevention of host cell death [39]. We therefore assume that the lifespan of infected cells is similar to that of uninfected monocytes, which have *in vivo* lifespan estimates ranging from one to two months [40]. Our minimum estimate of *δ* yields a lifespan estimate of 0.2 days, more than two orders of magnitude shorter than these values. This suggests that there must be other processes besides background infected cell mortality which are responsible for viral decline during a dengue infection.

**Figure 1.**
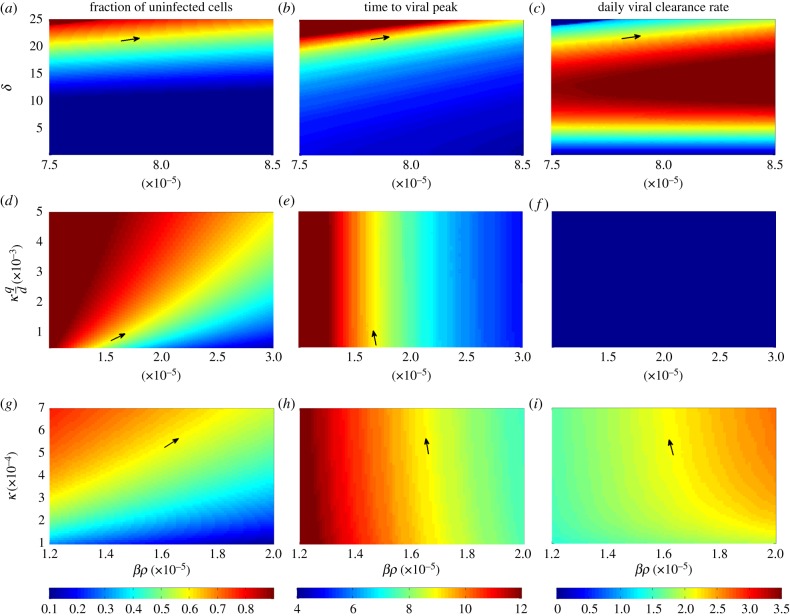
Primary infection model analysis. Rows show distinct within-host models examined; columns show the three characteristic features that the models seek to reproduce. The models are: (*a*–*c*) target cell limited model (equations (2.2)); (*d*–*f*) innate immune response model with fast NK-cell dynamics (equations (2.4)) and (*g*–*i*) innate immune response model with explicit NK-cell dynamics (equations (2.5)). The characteristic features are: (*a*,*d*,*g*) the final fraction of uninfected cells; (*b*,*e*,*h*) time to peak viraemia (in days) and (*c*,*f*,*i*) daily maximum viral clearance rate (in log_10_ copies ml^−1^ d^−1^). Each subplot shows the values of one characteristic feature as a function of two varied model parameters. Values in yellow meet the desired feature values of a primary dengue infection. Arrows point to the parameter values for which two or three characteristic features are simultaneously met. In subplots (*a–c*), *I*(0) = 1.4 × 10^−47^ cells ml^−1^. In subplots (*d*–*i*), *I*(0) = 8.62 × 10^−18^ cells ml^−1^. The target cell limited model meets all characteristic dengue features when *βρ* = 7.9 × 10^−5^ (cell ml^−1^)^−1^ d^−1^ and *δ* = 21.7 per day, although this high *δ* value is biologically implausible.

While this simple analysis indicates that a target cell limited model is unlikely to be able to reproduce the characteristic features of a primary dengue infection under biologically reasonable parameter values, we also performed a more systematic analysis to determine the model's ability to jointly reproduce the first three characterized features of a primary dengue infection (listed in §2.1). We do not include the fourth characteristic feature (peak viral load) because it is trivial to reproduce under the assumption of fast viral dynamics. Setting the initial number of susceptible cells (*S*(0)) to 3.5 × 10^5^ cells ml^−1^ based on absolute counts of monocytes from Green *et al*. [12], the model contains only three free parameters: the initial number of infected cells *I*(0), the infected cell clearance rate *δ*, and the parameter combination *βρ*. Fixing the value of *I*(0), we first examined whether there were values of *βρ* and *δ* that could jointly recover the three characterized features of a primary dengue infection (figure 1*a–c*).

This figure shows that for a biologically reasonable estimate of *δ* = 1/45 per day (corresponding to an approximate lifespan of 1.5 months), there is no value of *βρ* for which the model is able to recover the three characterized features of a primary dengue infection. Under unrealistically high values of *δ* (≈21.7 per day), however, the target cell model is able to recover all of the characterized features of a primary dengue infection (figure 1*a*–*c*). These results indicate that a simple model, extended to incorporate some process that aids in infected cell clearance, could in principle suffice in reproducing the key features of a primary dengue infection under biologically realistic parameter values. These overall results are robust to changes in the initial number of infected cells *I*(0), which impact the time to peak viraemia, but only minimally impact the other two viral dynamic features.

### 2.3. Models incorporating aspects of the innate immune response

Given the insufficiencies of the target cell limited model, we turn towards examining more complex within-host models, specifically those that include the innate immune response. Type I interferon (IFN) has been shown to be important in inhibiting viral replication and modulating downstream effects of the immune response, as well as activating natural killer (NK) cells [41], which play an important role in lysing virus-infected cells [42]. Specifically in the context of dengue, type I IFN levels have been shown to be elevated early in infection [10,43,44]. Similarly, early activation of NK cells has been demonstrated in dengue patients [12,45]. These studies suggest that these components of the innate immune response may play an important role in inhibiting dengue virus replication [46]. To model the effect of the innate immune response on dengue viral kinetics, we therefore start by considering the effects of IFN and NK cells as in a recently published influenza model [21]. This model makes the simplifying assumption that circulating NK cells are proportional to the level of circulating IFN. With this assumption, the innate immune response model is given by
2.3
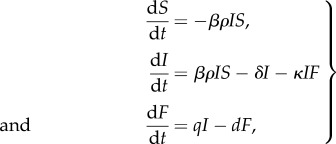

where IFN (*F*) is produced by infected cells and decays at rate *d*, and NK cells lyse infected cells at a rate proportional to *κ*.

Because IFN is known to have a short half-life [47], we further reduce the dimensionality of this model by assuming fast dynamics of *F*. With this quasi-steady-state assumption, equations (2.3) become
2.4
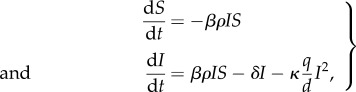

where *V* = *ρI* and *F* = (*q*/*d*)*I*. Our quasi-steady-state assumptions for free virus and IFN levels result in an expected linear relationship between *V* and *F*, consistent with the relationship observed in hospitalized DF patients experiencing a primary DENV-1 infection [44].

Assuming that *δ* = 1/45 per day, equations (2.4) have two free parameter combinations: *βρ* and *κ*(*q*/*d*), as well as initial condition *I*(0). For a given value of *I*(0), we analyse the effect of these parameter combinations on the first three characterized model features described in §2.1 (figure 1*d*–*f*). As in the case of the target cell limited model, peak viraemia levels are trivial to reproduce, so we do not consider this fourth feature.

This model can simultaneously recover the final fraction of uninfected cells (figure 1*d*) and the time to peak viraemia (figure 1*e*). However, it cannot simultaneously reproduce the known daily viral clearance rate (figure 1*f*) along with these other two features. In fact, the model cannot recover the daily viral clearance rate of 2.2 log_10_ copies ml^−1^ d^−1^ for any combination of *βρ* and *κ*(*q*/*d*) due to the nonlinearity introduced by the *I*^2^ term. The nonlinear viral decline (on the log_10_ scale) that results from the *I*^2^ term is also inconsistent with several viral kinetic studies, which show that viral clearance is largely linear on a log scale [9,10,29,48]. These findings indicate that equations (2.4) cannot jointly reproduce the features of a primary dengue infection. As for the target cell limited model, changes in the initial number of infected cells *I*(0) will not change these results as changes in *I*(0) only substantially impact the time to peak viraemia and not the other characterized model features.

We also considered a more complex model of IFN in which IFN is secreted according to the nonlinear, saturating term *qI*/(*K* + *I*) and decays at rate *dF* (see the electronic supplementary material). While this model is able to recover the characteristic features of a primary dengue infection, it includes unneeded complexity due to five free parameters and is only able to reproduce the viral clearance rate if IFN decays late in infection. This post-defervescent decline of IFN is inconsistent with IFN kinetic studies [10,44].

**Figure 2.**
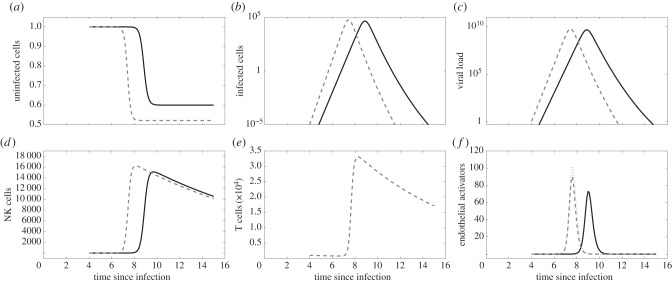
Within-host dynamics of primary and secondary dengue infections. Primary infection dynamics are shown in black solid lines; secondary infection dynamics are shown in grey dashed lines in subplots (*a*–*e*). (*a*) The fraction of uninfected cells (*S*(*t*)/*S*(0)) over the course of an infection, in days. (*b*) Infected cell dynamics (*I*(*t*); in cells ml^−1^). (*c*) Viral load dynamics (*V*(*t*); in copies ml^−1^). (*d*) NK-cell dynamics (cells ml^−1^). (*e*) T-cell dynamics (cells ml^−1^), secondary infection only. (*f*) The dynamics of endothelial activators (pg ml^−1^). For endothelial activator dynamics in dotted grey, it is assumed that endothelial activators are secreted by both infected cells and T cells (*α* = 2 × 10^−5^). For endothelial activator dynamics in dashed grey, it is assumed endothelial activators are only secreted by infected cells (*α* = 0).

Because equations (2.4) were unable to jointly recover the characteristic features of a primary dengue infection and the more complicated model considered in the electronic supplementary material could only reproduce the characteristic features under biologically implausible IFN dynamics, we next considered whether modelling NK cells explicitly would provide a sufficient minimal model. Relaxing the fast NK-cell dynamics assumption is a natural next step in increasing model complexity because both *in vivo* and modelling studies of human NK-cell dynamics during viral infection suggest that NK cells have a slow decay rate, and thus IFN dynamics are not representative of NK dynamics [22,49]. Furthermore, a study of dengue-infected patients showed that NK-cell activation markers were elevated 1 day after defervescence [12], a time point at which IFN-α levels are known to already have returned to low levels [10], suggesting that NK cells decay at a slower rate than IFN. As in [22], we therefore assume NK cells are activated at a rate proportional to IFN, *q*_N_*F*, and decay at rate *d*_N_*N*. With this assumption, the model is given by
2.5
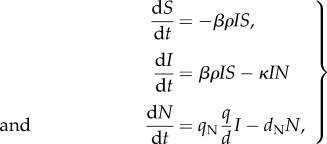

where *V* = *ρI*, *F* = (*q*/*d*)*I*, and we have assumed that *δ* ≈ 0. Based on [42,49], we assume NK cells have a half-life of 10 days, corresponding to a value of *d*_N_ of 0.07 per day. This model has three free parameter combinations (*βρ*, *κ*, and *q*_N_(*q*/*d*)) and one free initial condition *I*(0). Figure 1*g*–*i* shows how changes in *βρ* and *κ* impact the model features assuming given values of *q*_N_(*q*/*d*) and *I*(0). This figure indicates that a model that explicitly includes the dynamics of NK cells is able to recover the characteristic features of a primary dengue infection. Changes in *q*_N_(*q*/*d*) have similar effects on characteristic viral features as *κ*, and changes in *I*(0) again only affect the time to peak viraemia. Figure 2*a*–*d* shows the dynamics of uninfected cells, infected cells, free virus and NK cells for this model, parametrized to simultaneously reproduce the three features examined in figure 1, as well as the fourth feature of peak viraemia. Table 1 provides the specific parameter values used.

**Table 1.** Model parameters used for a primary and secondary dengue infection. Values of *I*(0) correspond to less than one infected cell to incorporate the time it takes for the first free virion to propagate an infection.

parameter	symbol	unit	primary infection	secondary infection	reference
initial number of target cells	*S*(0)	cells ml^−1^	3.5 × 10^5^	3.5 × 10^5^	[12]
initial number of infected cells	*I*(0)	cells ml^−1^	8.62 × 10^−18^	8.62 × 10^−18^	
initial number of NK cells	*N*(0)	cells ml^−1^	0	0	
initial number of T cells	*T*(0)	cells ml^−1^	—	1000	scales with *δ*_T_
initial number of endothelial activators	*E*(0)	pg ml^−1^	0	0	
infectivity rate	*β*	(copy ml^−1^)^−1^ d^−1^	1.54 × 10^−10^	1.85 × 10^−10^	
free virus production factor	*ρ*	copies cell^−1^	1.1 × 10^5^	1.1 × 10^5^	
kill rate of NK cells	*κ*	day^−1^	5.74 × 10^−4^	5.74 × 10^−4^	
kill rate of T cells	*δ*_T_	day^−1^	—	3 × 10^−5^	
recruitment rate of NK cells	*q*_N_(*q*/*d)*	day^−1^	0.52	0.52	
death rate of NK cells	*d*_N_	day^–1^	0.07	0.07	[42,49]
recruitment rate of T cells	*q*_T_	(cell ml^−1^)^−1^ d^−1^	—	1.2 × 10^−4^	
death rate of T cells	*d*_T_	day^−1^	—	0.1	[50]
recruitment rate of endothelial activators	*q*_E_	(pg ml^−1^) cell^−1^ d^−1^	0.01	0.01	
death rate of endothelial activators	*d*_E_	day^−1^	5	5

We note that the model recently published by Clapham *et al*. [25] also can reproduce the characteristic features of a primary infection. This model assumes that the immune response proliferates in response to interaction with infected cells, is present at low levels at the start of the infection and acts to kill infected cells. Although the authors do not explicitly interpret their model as one that models the cellular immune response, their immune response formulation closely mirrors previous model descriptions of T cells [51]. Contrary to this assumption, an experimental study in mice has shown that T cells are not critical for clearance of dengue virus during primary infection [3], though the relevance of mouse models in understanding human dengue infection has been questioned given the low replicative ability of dengue in mice. A study of T-cell responses in human primary dengue infections, however, appears to support the conclusions reached in the mouse study [52]: T cells are not activated in large quantities early in infection. Hence, T cells are unlikely to play a large role in clearing dengue virus in a primary infection. Since studies support the role of the innate immune response (over the adaptive immune response) in clearing primary dengue infections [3,46], we consider the NK model (equations (2.5)) as the minimal primary infection model.

### 2.4. Characteristic features of a secondary dengue infection

Because the dynamics of secondary dengue infections differ markedly from those of primary dengue infections, we here further seek to develop a minimal model capable of reproducing the characteristic features of such secondary infections. Relative to primary dengue infections, secondary dengue infections (encompassing all symptomatic clinical manifestations) are characterized by: (i) a shorter time to peak viraemia [9], (ii) a higher maximum viral clearance rate (approx. 2.8 log_10_ copies per millilitre per day for DF and 3 log_10_ copies per millilitre per day for DHF) [9], and (iii) a higher peak viraemia. We do not include as a feature the final fraction of uninfected cells because the dynamics of uninfected cells in a secondary infection are not well characterized. We also do not attempt to quantify specific values for peak viraemia or the time to peak viraemia, because viraemia levels from patients with secondary dengue infection are generally already in decline by the time patients are admitted [9].

### 2.5. Modification of the minimal primary infection model for a secondary dengue infection

We first sought to determine whether the minimal model capable of reproducing the features of a primary dengue infection could reproduce the above features of a secondary dengue infection. To recover a secondary infection's shorter time to viral peak and higher level of peak viraemia, a 20% increase in the viral infectivity rate *β* was sufficient, resulting in a peak viraemia level of 9.8 log_10_ viral copies at 7.4 days post viral inoculation. This level and timing of peak viraemia is consistent with virological data from secondary DF and DHF patients [9]. This reparametrization can also be easily interpreted biologically in the context of a phenomenon known as antibody-dependent enhancement (ADE), which proposes that antibodies from a first infection cross-react with virus from a secondary infection, but lead to incomplete neutralization. The resulting partially neutralized immune complexes are thought to enhance viral entry into Fc-receptor bearing target cells, and, as a result of this increased viral infectivity, lead to higher viraemia [53,54]. While an increased viral infectivity rate could reproduce shorter times to peak viraemia and higher levels of peak viraemia, this change could not reproduce a higher maximum viral clearance rate of at least 2.8 log_10_ copies per millilitre per day.

### 2.6. Model incorporating the cellular immune response

Motivated by current understanding of the role that the adaptive immune system plays in a secondary dengue infection, we sought to determine whether extending the minimal model by implementing the effect of a cellular immune response would be sufficient in reproducing the higher viral clearance rate seen during secondary infections. With this extension, the model becomes
2.6
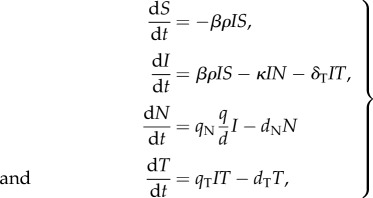

with, as before, *V* = *ρI*, *F* = (*q*/*d*)*I*. This model assumes that T cells, recruited at rate *q*_T_*IT* and decaying at rate *d*_T_*T*, lyse infected cells at rate *δ*_T_*IT*. Substituting *T*′ = *δ*_T_*T* into model equations (2.6) demonstrates that *T*(0) and *δ*_T_ cannot be estimated independently. We therefore set *T*(0) and examined the effect of changes in *δ*_T_ on model dynamics. We set *T*(0) to 1000 cells ml^−1^ since T cells undergo more than a 1000-fold expansion after a primary viral infection [55]. By assuming a lifespan of T cells of 10 days as in [50] and the same model parameters as for the primary infection model, with the exception of a 20% increase in *β*, the model retains only two free parameters: the T-cell recruitment rate *q*_T_ and the rate at which T cells lyse infected cells *δ*_T_.

**Figure 3.**
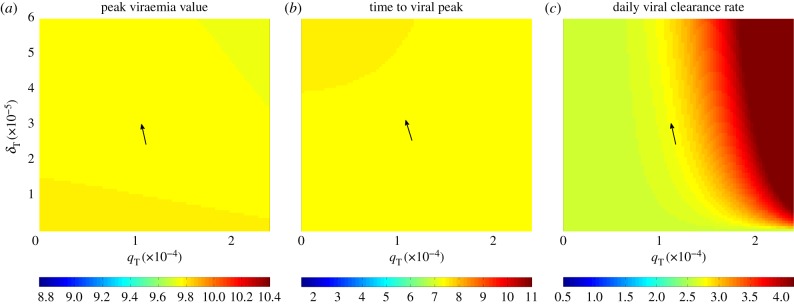
Secondary infection model analysis. The effect of changes in parameters *q*_T_ and *δ*_T_ on features of a secondary dengue infection using model (2.6): (*a*) peak viraemia value (log_10_ copies ml^−1^); (*b*) time to peak viraemia (days); and (*c*) daily maximum viral clearance rate (log_10_ copies ml^−1^ d^−1^). Values in yellow meet the desired feature values of a secondary dengue infection. Arrows point to the parameter values for which all characteristic features are met.

Figure 3 shows how these two parameter combinations affect the features of a secondary dengue infection. Both parameters have negligible effects on the peak viraemia value (figure 3*a*) and the time to peak viraemia (figure 3*b*), because T cells are not circulating at high levels before peak viraemia. However, higher values of both *q*_T_ and *δ*_T_ result in a higher viral clearance rate (figure 3*c*). Changes in *T*(0) mirror those of *δ*_T_: they influence the viral clearance rate but have little effect on the peak viraemia value and the time to peak viraemia.

These results indicate that all three features of a secondary infection can be recovered through a reparametrization of the viral infectivity rate *β* of the primary infection dengue model (reflecting the effect of the humoral immune response), along with the explicit incorporation of the cellular immune response. Figure 2 shows the dynamics of this model relative to the model dynamics for a primary dengue infection, with parameter values for these simulations provided in table 1. Intriguingly, these results are consistent with the recent statistical findings of Clapham *et al*. [25], which indicate that secondary dengue infections have higher estimated viral infectivity rates and higher viral clearance rates than do primary dengue infections.

## 3. Consistency of simulated model dynamics with observed virological data

**Figure 4.**
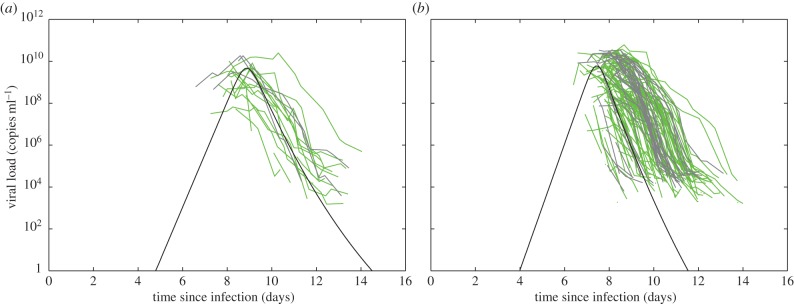
Simulations of the minimal models, parametrized according to table 1, alongside viral load data from DENV-1-infected adult patients [25]. (*a*) Primary infection data and model dynamics. Viral load data from primary infection DF patients (*n* = 15) are shown in green; viral load data from primary infection DHF patients (*n* = 3) are shown in grey. (*b*) Secondary infection data and model dynamics. Viral load data from secondary infection DF patients (*n* = 91) are shown in green; viral load data from secondary infection DHF patients (*n* = 32) are shown in grey. Model simulations recover generally shorter times to peak viraemia [9,56], higher viral clearance rates, [9,11] and higher peak viraemia levels [9,29] observed in symptomatic secondary infections relative to symptomatic primary infections.

Individual-level dengue viral load data have recently been made available [25], allowing us to compare our model simulations against DENV-1 dengue patient data. These data come from a clinical trial of adult dengue patients at the Hospital for Tropical Diseases in Ho Chi Minh City, Vietnam [9]. Viraemia was measured in the blood twice a day following hospital arrival, which was within 72 h of reported symptom onset. Virus was quantified by RT-PCR. Assuming an incubation period between viral inoculation and onset of symptoms of 6 days [35], figure 4 shows the viral load kinetics simulated using our minimal primary and secondary infection models under the parametrizations provided in table 1, along with data from both DF and DHF primary and secondary dengue infections. Though there is considerable variability in the observed data, the model simulations effectively capture their general dynamics. Furthermore, the data shown are consistent with the features described in §2.1 and 2.4.

The dengue viral load data contain both non-infectious and infectious viral particles [9]. However, we can consider only infectious virus in the model without impacting any of the other model variables by introducing a conversion factor that captures the ratio of all virus particles to infectious virus particles. By dividing the model parameter *ρ* by the conversion factor and multiplying the parameter *β* by this factor, the dynamics of the system remain unchanged, with the exception of the free virus dynamics. These dynamics are changed in that *V* is proportionally reduced by the magnitude of the factor at each time point, thereby modelling infectious virus particles instead of all virus particles.

## 4. Consistency of models with an increased risk of disease in heterologous secondary infections

Because DHF/DSS is characterized by increased vascular leakage [14], many studies have examined the relationship between pro-inflammatory cytokines that enhance capillary permeability and the risk of developing severe disease. Vascular leakage, the hallmark of DHF, occurs around the time of defervescence and lasts for approximately 48 h [57]. During this period, levels of TNF-α, IL-6 and IL-8 are consistently seen to be elevated in DHF patients compared to DF patients or healthy controls [57–60]. TNF-α in particular has been shown to be correlated with disease severity in numerous studies [14,31,57,61–64]. Furthermore, human polymorphisms that predispose for high TNF-α production have been shown to be associated with increased susceptibility to DHF [65]. IL-6 and IL-8 work synergistically with TNF-α to mediate activation of endothelial cells [14]. Although the complete role these cytokines play in dengue pathogenesis is not clear, their peak levels have been shown to be strongly correlated with disease severity [30,31,33].

The mechanisms that result in this high cytokine production are complex and most probably multifactorial [14,66]. Many studies have shown that viraemia levels are consistently correlated with disease severity [9,29,48]. As high viraemia is both a cause and a consequence of a high number of infected cells, and infected cells secrete cytokines, it has been suggested that high viraemia (reflecting high numbers of infected cells) is necessary for the development of plasma leakage [29,67]. However, high viraemia may not be sufficient for the development of plasma leakage [67].

T-cell-secreted cytokines have also been implicated in the development of severe disease [7,66,67]. Specifically, a theory known as original antigenic sin (OAS) proposes that memory T-cell populations with low avidity for the infecting virus of a heterologous secondary infection cause a strong pro-inflammatory response, as would any memory T-cell population activated by a secondary infection. Yet, because of their low avidity, the ability of these T cells to lyse infected cells, and thereby to protect against viral infection, may be poor [7,68]. In support of OAS, a study of dengue virus-specific T-cell responses in Thai children showed that the majority of dengue virus-specific T cells were of low affinity for the infecting serotype but had high affinity to one of the other three serotypes [68]. This study also showed that patients with DSS had stronger T-cell responses than individuals with less severe disease. In contrast to these pathologic effects of T cells, recent studies have suggested that T cells may instead play a protective role against severe disease [27,28,66]. Specifically, Weiskopf *et al*. showed that individuals with a strong T-cell response were associated with an HLA allele that appears to confer protection from severe disease [28]. They further showed that in transgenic mice, heterologous secondary infections responses directed to serotype-specific epitopes were impaired, but responses to conserved epitopes were not, suggesting that heterologous secondary infections are associated with a limited T-cell response [69]. Dung *et al*. [27] further documented a temporal mismatch between CD8^+^ T-cell activation and vascular leakage, suggesting that T-cell-secreted cytokines do not contribute to the development of severe disease.

Taken together, these studies show that while both infected cells and T cells are known to secrete cytokines associated with severe disease, the contribution of T-cell produced cytokines to severe disease is unclear [13,14,66]. To determine whether the minimal models developed above for a primary and a secondary dengue infection are able to recover the well-established pattern of increased risk of severe disease in a heterologous secondary infection, we consider a disease severity formulation under two different parametrizations. The first parametrization assumes that T-cell-secreted cytokines do not impact the development of severe disease, whereas the second assumes that T cells may act to exacerbate disease severity (although they cannot alone precipitate severe disease).

To keep the model as simple as possible, we group endothelial cell activating cytokines (TNF-α, IL-6, IL-8) into a single variable, *E*. We assume that these cytokines are primarily secreted by infected cells at rate *q*_E_*I*, and decay at rate d_E_*E*. To include the possible pathologic role of T cells, we assume that T-cell-secreted cytokines can amplify a pro-inflammatory response orchestrated by signaling between infected cells, as suggested by Dung *et al*. [27]. Because peak levels of endothelial cell activating cytokines have been shown to strongly correlate with disease severity [30,31,33], we assume that the risk of developing severe disease is proportional to the peak level of *E* (disease severity ∝ max(*E*)). Mathematically, the dynamics of disease-associated cytokines are therefore given by
4.1
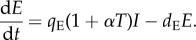

With *α* = 0, T-cell-secreted cytokines do not contribute to disease severity, whereas with *α* > 0, these cytokines exacerbate disease severity.

Figure 2*f* shows that both disease severity parametrizations for a secondary heterologous infection result in higher peak values of endothelial activators compared with a primary dengue infection, and therefore both parametrizations result in a higher risk of developing severe disease compared with a primary infection.

## 5. Consistency of models with virological indicators of disease severity

Clinical studies have indicated that viral kinetic features of an infection may be useful in predicting the risk of an individual developing severe disease [9,11,29,48]. These virological indicators include the level of peak viraemia, the time to peak viraemia, and the viral clearance rate. To determine whether the minimal models developed in the previous sections are able to reproduce the established relationships between these virological indicators of disease severity and the risk of developing severe disease, we used a Latin Hypercube Sampling (LHS) approach [70,71]. LHS is a random sampling method where uncertainty in each model parameter is considered in its contribution to a specified outcome, in our case, the risk of developing severe disease, which we take to be proportional to the peak level of endothelial activators *E*.

**Figure 5.**
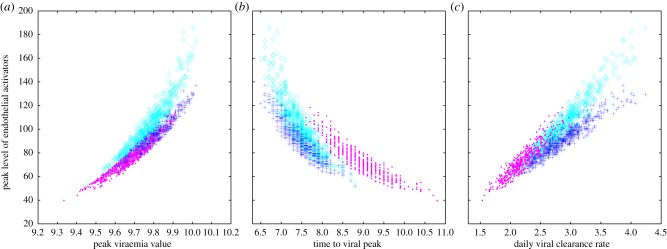
The relationship between virological indicators and the risk of developing severe disease. *S*(0), *β*, *ρ*, *κ*, *q*_N_(*q*/*d*) and *d*_N_ are varied for primary and secondary infections. *T*(0), *δ*_T_, *q*_T_ and *d*_T_ are additionally varied for a secondary infection. Primary infection simulations are shown with pink dots. Secondary infection simulations are shown with dark blue pluses (*α* = 0) and light blue diamonds (*α* = 2 × 10^−5^). (*a*) A scatterplot of peak viraemia (log_10_ copies ml^−1^) and the maximum value of endothelial activators (pg ml^−1^) for each LHS simulation. (*b*) A scatterplot of time to viral peak (days) and the maximum value of endothelial activators (pg ml^−1^) for each LHS simulation. (*c*) A scatterplot of daily viral clearance rate (log_10_ copies ml^−1^ d^−1^) and the maximum value of endothelial activators (pg ml^−1^) for each LHS simulation.

We first performed LHS simulations by varying parameters of both the primary infection and the secondary infection models (figure 5). For a primary infection, we considered variation in the initial condition *S*(0), and the model parameters *β*, *ρ*, *κ*, *q*_N_(*q*/*d*) and *d*_N_ on the peak level of endothelial activators. For a secondary infection, we considered variation in these same parameters and initial conditions as well as in the initial condition *T*(0) and the T-cell parameters *δ*_T_, *q*_T_ and *d*_T_ (figure 5). We used a sample size of 500 LHS simulations and sampled each parameter from a uniform distribution that extended ±10% from the point estimates provided in table 1.

### 5.1. Peak viraemia as a virological indicator

The first virological indicator of disease severity we consider is peak viraemia. A higher magnitude of viral load early in infection has been repeatedly associated with a higher risk of developing severe disease [9,10,29,48,56]. When a viral peak is observed, higher values of peak viraemia are associated with a higher risk of developing severe disease [9]. Figure 5*a* shows that our models reproduce the observed positive association between peak viral load and peak endothelial cell activator levels. Furthermore, figure 5*a* shows that during a secondary infection, both peak viral load and peak *E* levels (indicative of the risk of developing severe disease) are generally higher than in a primary infection. When we assume T-cell-secreted cytokines contribute to disease severity (*α* > 0), secondary infections with similar peak viraemia values to a primary infection nevertheless carry a higher peak level of endothelial activators.

Despite showing a clear positive relationship between peak viral load and the peak level of endothelial activators, figure 5*a* also indicates that this relationship is not perfect, that is, there is scatter. We can qualitatively understand this scatter between the level of peak viraemia and the peak level of endothelial activators by examining the directional effects of each varied parameter. In primary dengue infections, and in secondary dengue infections when disease severity is assumed to be due to only infected cell-secreted cytokines, the level of peak viraemia is almost a perfect predictor of disease severity (figure 5*a*). This is because increases in early acting parameters (*S*(0), *β*, *ρ*) all act to increase the number of infected cells, resulting in both higher peak viraemia and a higher maximum number of endothelial activators. Increases in late-acting parameters (*κ*, *q*_N_(*q*/*d*), *d*_N_, *T*(0), *δ*_T_, *q*_T_ and *d*_T_) all act to decrease the number of infected cells, resulting in both lower peak viraemia and a lower maximum number of endothelial activators.

In secondary dengue infections in which we assume that T-cell-secreted cytokines contribute to disease severity, we see more scatter in the relationship between peak viraemia and peak levels of *E*. This is because variation in parameters that affect T-cell dynamics now have disparate directional effects. Specifically, parameter changes that increase T-cell levels lower peak viraemia, but increase peak levels of *E*.

While our analysis suggests that peak viraemia is a relatively good indicator of disease severity, it also highlights that if T cells contribute significantly to the cytokine storm, the predictiveness of this indicator is limited.

### 5.2. Time to peak viraemia as a virological indicator

The second indicator of disease severity we consider is the time to peak viraemia. Several studies have shown that high viraemia levels early in infection are associated with a higher risk of developing severe disease [9,10,56]. For primary infections, a shorter time to peak viraemia is associated with higher disease severity, while peak viraemia is rarely observed during a secondary infection, suggesting that virus peaks earlier in secondary infection relative to a primary infection [9]. Figure 5*b* shows that our models recover this described negative relationship between time to peak viraemia and the peak level of endothelial activators. In secondary infections, when we assume T cells contribute to disease severity, a given time to peak viraemia results in a substantially higher peak level of endothelial activators compared to when T cells only play a protective role.

The relationship between the time to peak viraemia and the peak level of endothelial activators displays considerably more scatter than that observed between peak viraemia and the peak level of endothelial activators (figure 5*b* versus 5*a*). This is because the late-acting parameters (*κ*, *q*_N_(*q*/*d*), *d*_N_, *T*(0), *δ*_T_, *q*_T_ and *d*_T_) do not significantly impact the time to peak viraemia, while their values can considerably impact peak endothelial activator levels. Increased scatter is present when T-cell-secreted cytokines contribute to the level of endothelial activators, because the number of T cells impacts the peak level of endothelial activators, while essentially having no effect on the time to peak viraemia.

Our analysis suggests that the time to peak viraemia may be of limited use as a virological indicator of disease severity if there is considerable variation in the immune response of individuals. If T cells contribute to disease severity, the time to peak viraemia becomes an even less effective indicator of disease severity. On the other hand, if individual variability is largely dominated by early acting parameters, we would expect the time to peak viraemia to be an effective indicator.

### 5.3. Viral clearance rate as a virological indicator

Studies have shown conflicting associations between the rate of viral clearance and the risk of developing severe disease. Wang and colleagues, for example, found that a lower viral clearance rate was associated with the development of DHF in a study of 26 individuals experiencing secondary dengue infection [48]. By contrast, Vaughn *et al*. [11] found that a higher viral clearance rate was associated with the development of DHF in a study of 51 individuals experiencing either a primary or a secondary infection. This latter study further showed that the viral clearance rate was significantly higher in secondary dengue infections than in primary dengue infections. Similarly, a third study of 248 individuals experiencing either a primary or a secondary infection [9] found a higher rate of viral clearance in secondary dengue infections than in primary dengue infections, along with a slightly lower rate of viral clearance in patients with DHF during the secondary infection, compared to those with DF.

Figure 5*c* shows that our models predict a positive relationship between the rate of viral clearance (quantified as the maximal daily viral clearance rate) and the peak level of endothelial activators for both primary and secondary infections. When we assume T-cell-secreted cytokines contribute to disease severity, a given viral clearance rate results in a substantially higher peak level of endothelial activators compared to when T cells only play a protective role.

Again, there is considerable scatter in the relationship between viral clearance rate and the peak level of endothelial activators. This is because changes in the parameters that affect NK- and T-cell dynamics have opposite effects on the viral clearance rate than on the level of endothelial activators. Parameter changes that increase the number of NK cells or T cells simultaneously increase the viral clearance rate while decreasing the number of infected cells, and therewith peak endothelial activator levels.

While our findings indicate that the viral clearance rate may not be a good indicator for predicting the onset of severe disease, they are helpful in understanding the conflicting results of the studies described above. The results from Wang *et al*. [48], showing that a lower viral clearance rate was associated with increased disease severity suggest that T cells in these patients played largely protective roles, such that a low clearance rate reflects a suboptimal T-cell response. The opposite result observed in the other two studies may be explained by inter-individual variation in early acting parameters, or, if T cells contribute to disease severity, by a strong T-cell response.

### 5.4. Robustness of virological indicator results to alternative disease severity formulations

In the electronic supplementary material, we consider whether the results shown in figure 5 are robust to alternative disease severity formulations. One alternative formulation reflects the assumption that T cells can secrete cytokines that contribute to disease severity, and that these secreted cytokines do not simply amplify, but contribute additively to peak *E* levels. This assumption leads to the alternative disease severity formulation of d*E*/d*t* = *q*_E_*I* + *aT* – *d*_E_*E* instead of equation (4.1), where *a* is a parameter quantifying the production rate of endothelial activators by T cells. This formulation reproduces the relationships between the virological indicators and peak levels of *E* observed with the original disease severity formulation provided in equations (4.1) (electronic supplementary material, figure S2). With this alternative formulation, however, an individual with high T-cell counts but low viraemia can experience a high risk of developing severe disease, a pattern that is not supported by the literature [29,67].

Returning to equation (4.1) to describe endothelial activator dynamics, we can alternatively consider the risk of severe disease to be proportional to the total amount of endothelial activators secreted during the infection (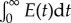). This alternative formulation also reproduces the described relationships between the virological indicators and the risk of developing severe disease (electronic supplementary material, figure S3). However, this disease measure incorporates cytokine dynamics after vascular leakage has occurred, making it, to some degree, biologically unreasonable. The incorporation of post-defervescence cytokine dynamics also generates more scatter in the relationships between the virological indicators and the risk of developing severe disease.

## 6. Discussion

Here we have developed a suite of within-host mathematical within-host dengue models to describe the dynamics of the virus and to better understand the development of severe dengue disease. By first beginning with a target cell model, and adding increasing complexity, we have arrived at minimal models able to reproduce characterized viral features for both primary and secondary infections. We have shown that only the innate immune response is needed to recover the features of a primary dengue infection, and that for a secondary dengue infection, a higher rate of viral infectivity (representing ADE) is needed to recover the higher peak viraemia value and the shorter time to peak viraemia, while T cells are needed to recover the higher viral clearance rate. We parametrized our minimal models using a combination of literature estimates and described features.

Our models are the first dengue models to link within-host dynamics to a quantitative measure of disease severity. Specifically, we assumed that severe dengue disease, characterized by vascular leakage, results from a cytokine storm, consistent with current thinking [7,13,59]. We developed two disease severity formulations differing in the contribution of T-cell-secreted cytokines to disease severity. While these disease severity formulations greatly simplify the complex mechanisms leading to severe dengue disease, they are a first step in providing a quantitative framework for understanding the processes jointly regulating viral dynamics and driving the onset of severe disease.

We have shown through simulation that the models reproduce associative relationships between known virological indicators and the peak level of endothelial activators, our measure of the risk of developing severe disease, and that these relationships are robust to our disease severity parametrizations. However, we have also shown that the effectiveness of these indicators depends heavily on the (as yet disputed) contribution of T cells in precipitating severe disease.

Although our models can reproduce viral patterns and the association between known virological indicators and the risk of developing severe disease, our current parametrizations of the models are not obtained through statistical fits to data. Instead, our parametrization is based on well-established viral features. While statistical fits to data, as performed by Clapham *et al*. [25], are extremely valuable, the lack of available viral and immunological data early in dengue infection is currently a limiting factor in robustly estimating model parameters. This underscores the importance of large longitudinal cohort studies in facilitating the collection of these types of data. The Pediatric Dengue Cohort Study in Nicaragua [72], for example, routinely tests children for dengue, and detects inapparent as well as symptomatic infections. This type of study should be able to obtain detailed viral measurements early in infection, specifically before viraemia peaks. Along with viral kinetic data, more detailed kinetic data of NK- and T-cell activation markers in relation to the timing of vascular leakage, the hallmark of DHF, are needed to help elucidate the extent of the protective role of NK cells and the contribution of T cells to disease severity. Additionally, more detailed kinetic studies of cytokines in relation to the timing of vascular leakage will be useful in determining which cytokines are the best markers of disease severity.

In our parametrization of the models, we focused exclusively on the differences between a primary and a secondary dengue infection. However, it is well known that the risk of developing severe disease in a secondary dengue infection can depend on the identities of the particular virus serotype of the primary infection as well as of the virus serotype of the secondary infection [53,73]. The secondary infection model is sufficiently flexible to be able to examine this order-of-infection effect simply through re-parametrization. A thorough analysis, however, will critically again depend on data availability. We are currently unaware of any studies that examine viral load dynamics in individuals experiencing a secondary dengue infection in which the serotype identity of the primary infection has been documented.

We also did not explicitly address temporary cross-protection or cross-immunity between serotypes with our model, although both observational and epidemiological studies have indicated that individuals are protected either from infection or disease for up to 2 years following a primary infection [74–76]. Our models, however, could easily accommodate this effect through a re-parametrization. Presumably, individuals are protected in a heterologous secondary infection for a period following a primary infection, because protective antibody levels remain sufficiently high to be capable of effectively neutralizing virus. In our model, this would lead to an increase in the viral clearance rate, lowering *ρ* and the within-host basic reproduction number. Because decreases in the basic reproductive number result in either the inability for a virus to infect an individual or reduced viraemia, this simple re-parametrization of the model indicates that we should expect to see a lower risk of developing severe disease in recently infected individuals.

While alternative parametrizations can be used to consider certain questions we have not examined here, our models make several simplifying assumptions that need to be relaxed to address other outstanding questions. First, the secondary infection model assumes that the humoral immune response occurs immediately and stays at a constant level (represented by an increased *β*). Because our current model does not explicitly include the dynamics of the humoral immune response, we may be oversimplifying the contribution of antibodies to disease severity. Furthermore, a dynamic adaptive immune response needs to be modelled to understand, for example, the downstream effects of the antibody response in modulating cytokine production (intrinsic ADE) [77]. Second, for simplicity, we do not distinguish between different types of cytokines and therefore do not include the roles of certain cytokines in upregulating or downregulating the production of other cytokines, which may be important in understanding the complex mechanisms leading to the occurrence of vascular leakage [14,59].

Although the assumptions that our within-host mathematical models currently make limit their applicability to certain questions, we believe that they stand as a good jumping off point for more complex models. They are the first attempt to understand how the dynamics of dengue virus and the immunological response of its host affect the risk of developing severe disease. They provide insight into the contribution of infected cells and T cells to disease severity and highlight the importance of within-host dynamics early in the infection in predicting disease severity. They also highlight that the predictiveness of virological indicators are dependent on our understanding of the mechanisms leading to severe disease. Modifications of the model, in terms of both parametrization and structure, will require more extensive datasets and, critically, continued collaborative interactions between quantitative biologists and dengue virologists and immunologists.

## Supplementary Material

Minimal within-host dengue models highlight the specific roles of the immune response in primary and secondary dengue infections electronic supplementary material
