# Supplementary material for: Minimal within-host dengue models highlight the specific roles of the immune response in primary and secondary dengue infections
Source: J R Soc Interface. 2015 Feb 6;12(103):20140886. doi: 10.1098/rsif.2014.0886 (PMC4305404; doi:10.1098/rsif.2014.0886)
Supplement: Minimal within-host dengue models highlight the specific roles of the immune response in primary and secondary dengue infections electronic supplementary material [file rsif20140886supp1.pdf]

Minimal within-host dengue models highlight the specific roles of the immune response in primary and secondary dengue infections

Electronic supplementary material

By Rotem Ben-Shachar[1], Katia Koelle[2,3]

1. Program in Computational Biology and Bioinformatics, Duke University, Durham, NC, USA; 2. Department of Biology, Duke University, Durham, NC, USA; 3. Fogarty International Center, National Institute of Health, Bethesda MD, USA

1. Primary infection model with saturating production of IFN

Here we consider a more complicated model of IFN, namely one which uses Michaelis-Menten dynamics to describe the secretion of IFN as a function of infected cells. We again assume that IFN decays at some rate dF and that NK cells kill infected cells at a rate proportional to κIF. The model is given by:

$$\begin{aligned} dS/dt &= -\beta\rho IS \\ dI/dt &= \beta\rho IS - \delta I - \kappa IF \\ dF/dt &= \frac{qI}{I+K} - dF \end{aligned}$$

where $V = \rho I$. For simplicity, we also assume the rate of infected cell death $\delta = 0$. This model has five free parameter combinations ($\beta\rho, \kappa, q, d$ and K) and one free initial condition ($I(0)$). Supplementary figure 1 shows that this model (under the parameterization provided in Supplementary Table S1) can recover all of the characteristic features of a primary dengue infection: the final fraction of uninfected cells, time to peak viremia, viral clearance rate, and peak viral load. To reproduce these features, particularly the high viral clearance rate, the model requires high IFN levels late in infection (Figure S1d). These high IFN levels late in infection are, however, inconsistent

with kinetic studies that have shown that IFN-α levels of dengue patients peak early on in infection (before or on the first few days of symptoms) [1] and have decayed to very low levels by defervescence [2].

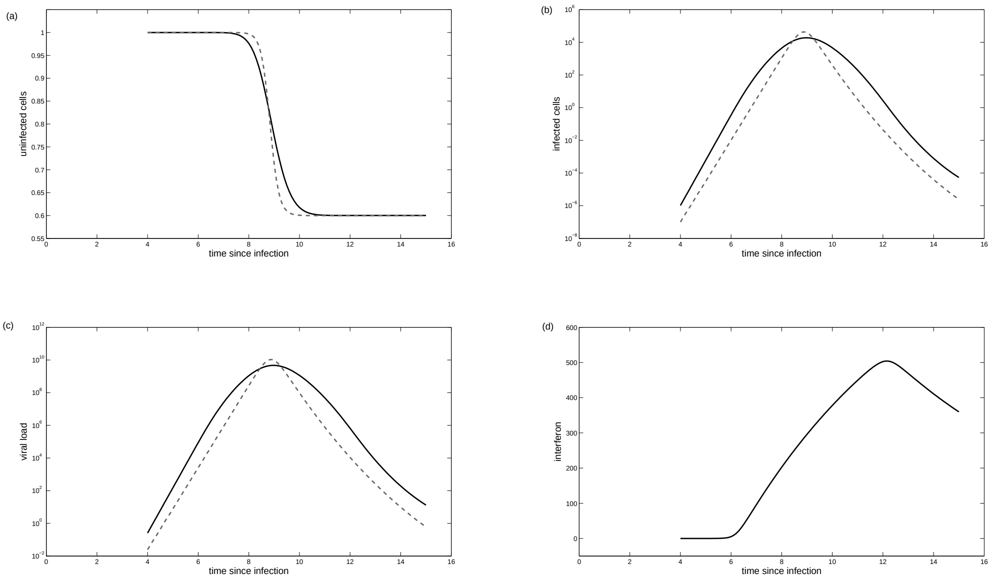

Supplementary Figure S1. Simulated within-host dynamics of a primary infection. Primary infection dynamics using the saturating IFN production model are shown in solid black; primary infection dynamics using equations 2.5 are shown in dashed gray. (a) The fraction of uninfected cells over the course of an infection ($S(t)/S(0)$). (b) Infected cell dynamics ($I(t)$ cells/mL). (c) Viral load dynamics ($V(t)$ copies/mL). (d) IFN dynamics for the saturating IFN production model. IFN dynamics for equations 2.5 are not shown, but follow proportionally to infected cell dynamics.

Supplementary Table S1. Model parameters for a primary dengue infection using the saturating IFN production model. All parameters not listed are the same as listed for a primary infection in Table 1 in the main text.

Parameter	Symbol	Unit	Primary Infection
Initial amount of IFN	$F(0)$	pg/mL	0
Infectivity rate	β	$(\text{copy/mL})^{-1}$ day^{-1}	7.38×10^{-11}
Free virus production factor	ρ	copies cell^{-1}	2.5×10^{5}
Kill rate of IFN	κ	$(\text{pg/mL})^{-1}$ day^{-1}	0.02
Recruitment rate of IFN	q	day^{-1}	127
Death rate of IFN	d	day^{-1}	0.13
Saturation constant	K	cells/mL	1.17

2. Alternative disease severity formulations

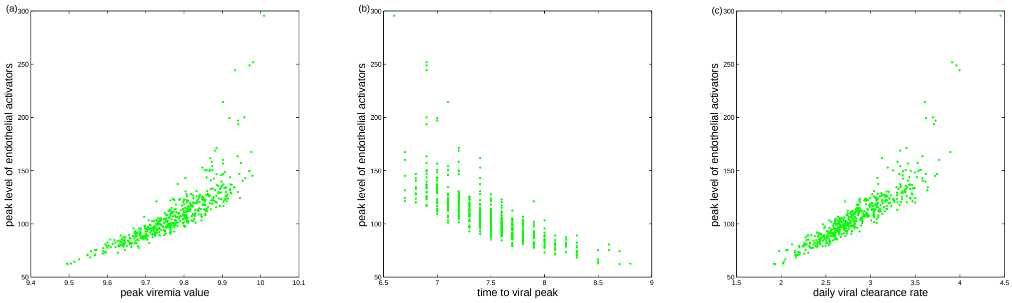

Supplementary Figure S2. The relationship between virological indicators and the risk of developing severe disease in a secondary dengue infection when T-cells contribute additively to the risk of severe disease. Here, the dynamics of endothelial activators are given by $dE/dt = q_E I + aT - d_E E$ where $a = 1 \times 10^{-2}$. $S(0), \beta, \rho, \kappa, q_N \frac{q}{d}$, d_N, $T(0), \delta_T, q_T$, and d_T are varied in these LHS simulations. (a) A scatterplot of peak viremia ($\log_{10}$ copies/mL) and the peak level of endothelial activators for each LHS simulation. (b) A scatterplot of time to viral peak (days) and the peak level of endothelial activators for each LHS simulation. (c) A scatterplot of daily viral clearance rate ($\log_{10}$ copies/mL) and the peak level of endothelial activators for each LHS simulation.

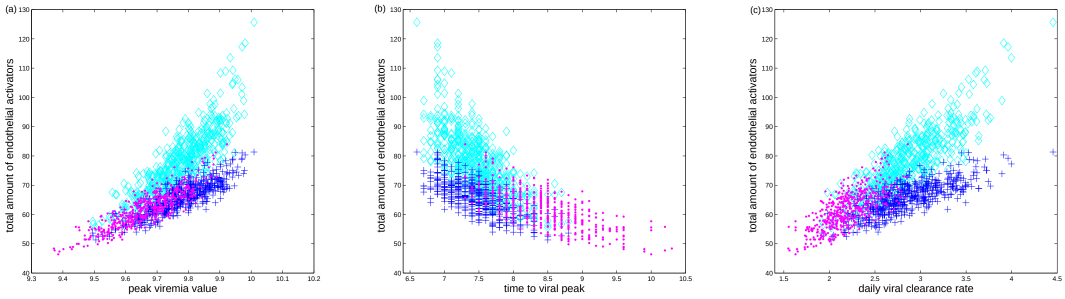

Supplementary Figure S3. The relationship between virological indicators and the risk of severe disease. Here, the risk of severe disease is assumed to be proportional to the total amount of endothelial activators. $S(0), \beta, \rho, \kappa, q_N \frac{q}{d}$ and d_N are varied for a primary and secondary infection. $T(0), \delta_T, q_T$, and d_T are additionally varied for a secondary infection. Primary infection simulations are shown with pink dots. Secondary infections simulations are shown with dark blue pluses ($\alpha = 0$) and light blue diamonds ($\alpha = 2 \times 10^{-5}$). (a) A scatterplot of peak viremia ($\log_{10}$ copies/mL) and the risk of severe disease for each LHS simulation. (b) A scatterplot of time to viral peak (days) and the risk of severe disease for each LHS simulation. (c) A scatterplot of daily viral clearance rate ($\log_{10}$ copies/mL) and the risk of severe disease for each LHS simulation.

References

[1] Tang, Y., Kou, Z., Zhang, F., Yao, X., Liu, S., Ma, J., Zhou, Y., Zhao, W., Tang, X. & Jin, X., 2010 Both viremia and cytokine levels associate with the lack of severe disease in secondary dengue 1 infection among adult chinese patients. *PLoS One* **5**, e15631. (doi:10.1371/journal.pone.0015631).

[2] Libraty, D. H., Endy, T. P., Houng, H.-S. H., Green, S., Kalayanarooj, S., Suntayakorn, S., Chansiriwongs, W., Vaughn, D. W., Nisalak, A., Ennis, F. A. *et al.*, 2002 Differing influences of virus burden and immune activation on disease severity in secondary dengue-3 virus infections. *J Infect Dis* **185**, 1213–21. (doi:10.1086/340365).
